# Epigenetics—a potential mediator between air pollution and preterm birth

**DOI:** 10.1093/eep/dvv008

**Published:** 2016-01-12

**Authors:** Vania W. Lin, Andrea A. Baccarelli, Heather H. Burris

**Affiliations:** ^1^ Chicago Medical School at Rosalind Franklin University of Medicine and Science, North Chicago, IL, 60064 USA;; ^2^ Department of Neonatology, Beth Israel Deaconess Medical Center & Division of Newborn Medicine, Boston Children’s Hospital, Harvard Medical School, Boston, MA, 02215 USA;; ^3^ Department of Environmental Health, Harvard T.H. Chan School of Public Health, Boston, MA, 02115 USA

**Keywords:** air pollution, preterm birth, epigenetics, DNA methylation, pregnancy

## Abstract

Preterm birth is a major cause of infant morbidity and mortality and a potential risk factor for adult chronic disease. With over 15 million infants born preterm worldwide each year, preterm birth poses a global health concern. There is a possible association between air pollution and preterm birth, though studies have been inconsistent, likely due to variation in study design. How air pollution induces health effects is uncertain; however, studies have repeatedly demonstrated the effects of air pollution on epigenetic modifications. More recent evidence suggests that epigenetics may, in turn, be linked to preterm birth. Discovery of environmentally modifiable epigenetic processes connected to preterm birth may help to identify women at risk of preterm birth, and ultimately lead to development of new preterm birth prevention measures.

## Introduction

Preterm birth, defined as birth before 37 completed weeks of gestation, is a major cause of infant morbidity and mortality [ 1 ] and a potential contributor to adult chronic disease [ 2 ]. Approximately 15 million infants are born preterm annually worldwide [ 3 ], with half a million in the United States alone [ 4 ]. Air pollution is one of many potential risk factors for preterm birth. Air pollutants are produced from many sources, including wood burning, coal burning, dry cleaning, motor vehicle exhaust, and industrial emissions [ 5 ]. The most commonly surveyed criteria air pollutants include particulate matter ≤2.5 µm in aerodynamic diameter (PM _2.5_ ) and ≤10 µm in aerodynamic diameter (PM _10_ ), carbon monoxide (CO), oxides of nitrogen (NO _x_ ), sulfur dioxide (SO _2_ ), and ozone (O _3_ ) [ 6–8 ].

Results from studies on the effects of air pollution on preterm birth vary greatly [ 9 ]. Differing results between studies may be due to a variety of factors, including misclassification arising from both exposure and outcome ascertainment errors [ 10–12 ] and the heterogeneity of exposure and outcome assessment [ 7 , 11 , 12 ]. However, the biological plausibility of air pollution as a risk factor for preterm birth may be inferred through the well-established connection between air pollution and a number of other inflammatory disease processes [ 13–15 ]; preterm birth may occur as a result of inflammation that presents in several pathophysiologic states, including infection, preeclampsia, and smoking [ 16 ]. Furthermore, given the similarity of chemical composition between air pollution and tobacco smoke and the strong evidence for the association between maternal smoking and preterm birth [ 17 ], air pollution remains a likely risk factor for preterm birth.

In considering the mechanisms by which air pollution may exert its effects, epigenetics has become an increasingly likely candidate. Epigenetic processes include DNA methylation, histone modifications, and noncoding RNA expression [ 18 ], all of which affect gene expression without changing the underlying DNA sequence. Studies have shown that various environmental exposures, including air pollution, induce epigenetic changes associated with inflammation [ 19 ].

Given the connections between (i) air pollution and preterm birth and (ii) air pollution and epigenetics, it may be inferred that a link exists between epigenetics and preterm birth as well. A growing body of research has yielded findings associating DNA methylation and microRNA (miRNA) with preterm birth, largely by their involvement in inflammatory processes. Although results of epigenetic studies of preterm birth are not conclusive due to the relatively small number of studies and their limitations, the findings are promising and are worth further exploration. Practical applications of understanding the epigenetic marks that result from air pollution exposure and lead to risk of preterm birth may include opportunities to identify highly exposed individuals to reduce exposure and develop pharmaceutical interventions to mitigate risk.

### Air Pollution and Preterm Birth

As individual studies vary with respect to their findings on the effects of air pollution on preterm birth, a number of investigators have performed systematic reviews to determine whether an association exists. However, results of these systematic reviews are contradictory. Some reviewers have deemed the link between air pollution and preterm birth to be inconclusive, pending further research [ 6 , 8 , 9 , 20 , 21 ]. In contrast, other reviewers reported significant associations. Shah and Balkhair [ 7 ] found associations between PM _2.5_ , SO _2_ , and preterm birth; however, the effects of PM _10_ , CO, NO _x_ , and O _3_ were inconclusive. Similarly, Ferguson *et al* . [ 11 ] reported that PM _2.5_ and SO _2_ , along with environmental tobacco smoke (ETS) and polycyclic aromatic hydrocarbons (PAHs), were associated with preterm birth but were unable to draw conclusions on the effects of the remaining criteria air pollutants. In a pooled analysis of odds ratio (OR) estimates for preterm birth, Stieb *et al* . [ 22 ] concluded that estimates for third trimester exposures were more consistent and precise compared to those for other periods of exposure, likely due to lower heterogeneity (as measured by the *I*^2^ value) among third trimester studies. For third trimester exposures, only the OR estimates for PM _10_ and CO were significant: per 1 ppm CO, OR = 1.04 [95% confidence interval (CI) = 1.02–1.06], while per 20 µg/m ^3^ PM _10_ , OR = 1.06 (95% CI = 1.03–1.11). The authors found no significant results for PM _2.5_ , NO _2_ , and O _3_ and provided no estimate for SO _2_ . Ghosh *et al* . [ 23 ] stated that fetal sex affected the association between air pollution and preterm birth, with exposed male fetuses at higher risk of preterm birth compared to exposed female fetuses.

#### Potential Causes of Inconclusive Results

While an association between air pollution and preterm birth seems probable, a number of issues affect the interpretation of research findings. For studies with smaller sample sizes, lack of power may limit the detection of subtle associations [ 24 ]. A major cause of inconclusive results, however, is likely misclassification.

For studies on air pollution and preterm birth, exposure assessment is particularly vulnerable to misclassification. Air quality monitoring data, commonly used by investigators to assess exposure, are affected by this bias, as the collected information does not account for the fluctuations in exposure that are dependent on daily activities (i.e. time–activity patterns). In birth outcome studies, researchers use birth certificates to derive information on birth outcomes and participant characteristics, including the residential address at the time of delivery. The residential address is then used to locate the closest air quality monitoring station, and the data from this station are used to estimate the participant’s exposure to air pollution.

However, the use of residential address to locate the nearest air quality monitoring station and estimate air pollution exposure may introduce exposure misclassification. Participants likely travel to several locations throughout the day and may spend more time at locations other than their residential address (e.g. workplace). Furthermore, the residential address at the time of delivery may not be the same as the one at the time of pregnancy. The resultant exposure misclassification may bias the results toward the null hypothesis [ 25 ]. Nonetheless, it should be noted that ground level monitors better capture temporal variability in a locale than land-use regression models, which more effectively capture spatial variability. Thus, ground monitoring may be more suited than other methods to reflect a participant’s exposure in specific time periods during pregnancy in her general vicinity of work and home.

One method to reduce misclassification of exposure is the use of personal air quality monitors. These monitors improve accuracy by accounting for time-activity patterns. However, there are several potential issues associated with this method of exposure assessment. First, use of personal monitors is substantially more costly than the use of local air quality monitors and may limit the sample size, thus causing the study to be potentially underpowered for the detection of subtle associations. Second, the accuracy of the data may be reduced depending on whether the participants are compliant with wearing the monitors at all times. Finally, selection bias may affect the study if some women are more likely to participate than others given the added burden of wearing personal air quality monitors (e.g. women who are solely interested in financial compensation or women who are genuinely invested in the research); women who participate in the study may have substantially different levels of exposure to air pollution and different inherent risks for the outcome of preterm birth from those who do not. As such, although personal air quality monitors yield more accurate exposure data, they do not address all of the issues affecting studies on air pollution and health effects.

Another method of addressing misclassification of exposure is the use of biomarkers. Metabolite concentrations may reflect more accurately the level of exposure than air quality monitoring data. However, like air quality monitors, biomarkers do not distinguish between routes of exposure [ 12 ]. For example, exposure to PAHs may occur through ingestion of charbroiled foods or inhalation of ambient air. Investigators may misclassify participants on the basis of biomarker levels, as these levels may reflect exposure to PAHs from ingestion rather than inhalation, the exposure route of interest [ 11 ].

Additionally, biomarkers are costly and labor-intensive to collect compared to population-level data such as local air quality monitoring data. Researchers must also specify the target substance to measure and determine the appropriate biomarker. Furthermore, the relationship between exposure and metabolite production is important to consider in determining the timing of the exposure, as metabolites differ in their half-lives [ 12 ]. These aspects should be taken into account when using biomarkers to assess exposure.

Heterogeneity of exposure assessment is another major factor that may affect the interpretation of results. Studies not only vary with respect to doses of exposure but also vary in their method of air pollution ascertainment, with common methods including use of air quality monitors, country-wide averages, distance-weighted averages, and county-wide averages [ 7 ]. While some of these methods offer information on specific pollutants (e.g. air quality monitoring data), others do not (e.g. distance to roadway exposure estimates). Thus, the accuracy of the data in reflecting the true exposure of participants may fluctuate from study to study depending on the chosen method.

In addition, data on air pollutants may be presented as composite exposures rather than data specific to each pollutant. This commonly occurs when presenting data on pollutants originating from the same source, such as CO and NO _x_ from motor vehicle exhaust and PM and SO _2_ from industrial emissions [ 12 ]. As such, because of variations in the presentation of air pollution data, studies may differ greatly in the kind of exposure data utilized, even when involving the same target pollutants, making comparison across studies difficult.

Studies may differ in how they account for exposure throughout gestation. While some investigators break down the exposure data by each trimester, others examine the average exposure over the entire pregnancy. This latter method may result in missed capture of effect, as the association between air pollution and preterm birth may change depending on the timing of exposure. Ritz and Wilhelm [ 12 ], in their review of literature on the association between air pollution and preterm birth, stated that the differences in effect of exposure between different gestational stages may indicate periods of susceptibility during which the fetus has increased sensitivity to the effects of air pollution. In particular, the first and third trimesters are likely susceptible windows; implantation takes place in the first trimester, while substantial fetal growth occurs during the third trimester [ 7 ]. The interruption of these crucial processes may lead to adverse outcomes, including preterm birth.

The use of advanced statistical methods may address the issue of windows of vulnerability. Distributed lag models may help delineate windows of vulnerability for preterm birth as they have for epidemiologic studies on air pollution and death [ 26 ]. Additionally, the use of time-to-event or accelerated failure time models may be of use when studying gestational age, given that gestational age is not normally distributed and is abruptly truncated at 42 or 43 weeks of gestation [ 27 ].

Heterogeneity of outcome assessment with respect to preterm birth may also affect whether investigators find an association between air pollution and preterm birth. Researchers vary in examining preterm birth as a uniform outcome of birth at less than 37 weeks of gestation or as more detailed categories of very preterm birth, moderate preterm birth, and late preterm birth—the definitions of which may differ between studies. Additionally, most studies do not differentiate between spontaneous and medically indicated preterm births, often because such details are not included or are not reliable at a population level (e.g. birth certificate data). These outcomes may further be divided into their subtypes: preterm labor, cervical incompetence, placental abruption, and preterm premature rupture of membranes for spontaneous preterm births and preeclampsia and poor fetal growth for medically indicated preterm births [ 16 ]. Various types of preterm birth may have different associations with air pollution; by studying an aggregate outcome of preterm birth instead of examining specific types, investigators may not capture these associations [ 11 ].

#### Biological Plausibility

Despite the variation in study results, the association between air pollution and preterm birth remains biologically plausible. Specifically, the effects of inflammation and the analogous exposure of tobacco smoke, both clearly associated with preterm birth, lend support to the concept of air pollution as a risk factor for preterm birth.

Kannan *et al* . [ 28 ] described the various biological pathways through which air pollutants induce systemic oxidative stress and inflammation and cause changes in blood coagulation, endothelial function, and hemodynamic responses. In particular, systemic oxidative stress and inflammation may play important roles in preterm birth. While increased production of inflammatory cytokines may be a normal preparatory step for term deliveries [ 29 ], the timing of increased production appears to be key in regards to the likelihood of term or preterm delivery. Abnormal early production of proinflammatory cytokines seems to be a potential factor in increasing the likelihood of preterm birth [ 12 ]. The strong evidence for associations between air pollution and conditions such as cardiopulmonary diseases [ 13 , 14 ] and type 2 diabetes mellitus [ 15 ], as mediated by inflammatory pathways, lends biological plausibility to the inflammatory mechanisms through which air pollution may affect preterm birth.

Ample evidence for the effects of maternal smoking on preterm birth imparts biological plausibility to the causal association between air pollution and preterm birth, as air pollution contains many of the same chemicals as tobacco smoke. In a meta-analysis of 20 studies, the pooled OR estimate for the outcome of preterm birth for any maternal smoking versus no maternal smoking was 1.27 (95% CI = 1.21–1.33). The authors also observed a dose–response relationship at low to moderate levels of smoking [ 17 ]. Among the many chemicals tobacco smoke contains, CO, PAHs, lead, and cadmium are included in suspected factors of preterm birth [ 5 , 12 ]. Studies have shown that both CO and PAHs are able to cross the placenta and directly affect the fetus [ 30–32 ], lending biological plausibility to the effects of tobacco smoke on fetal development. The adverse changes these chemicals induce in the fetus, including decrease in fetal weight, may lead to preterm delivery [ 33 ].

Results of studies linking maternal smoking to preterm birth suggest that ETS exerts similar effects. However, like air pollution research, studies on ETS and preterm birth are inconsistent in their results. One reason may be measurement error, particularly of exposure. Many studies rely on self-reported exposure to ETS; some women may be unwilling to disclose exposure or may not be aware of exposure [ 34 ]. Misclassification of exposure may thus result, and findings may be biased toward the null hypothesis [ 25 ]. Another reason may be the nature of ETS effects. As exposure to ETS is equivalent to approximately 1% of exposure to maternal smoking [ 35 ], ETS effects may be subtle, thus requiring large sample sizes for detection. However, the similarities in the chemical compositions of ETS and tobacco smoke support the biological plausibility of the association between ETS and preterm birth and encourage further investigation.

### Air Pollution and Epigenetics

Although a connection between air pollution and preterm birth, potentially mediated by inflammation, appears to be biologically plausible, the exact mechanism by which air pollution exerts its effects remains unclear. Recent studies suggest that epigenetics may be the link between air pollution and health effects. Epigenetic processes affect gene expression, thus inducing changes in biological function without modifying the underlying DNA sequence. While most investigators have focused on DNA methylation, more recently others have examined histone modifications and noncoding RNA expression.

#### DNA Methylation

DNA methylation typically regulates gene expression via inhibition of DNA transcription [ 36 ] and silencing of DNA repetitive sequences and transposons [ 37 , 38 ]. The methylation of repetitive elements, namely long interspersed nuclear element-1 (LINE-1) and Alu, provides a summary measure of thousands of sequences across the genome [ 39 ]. Decreased DNA methylation of repetitive elements and associated inflammation and cellular stress have been implicated in disease processes [ 40 ], including cardiovascular and respiratory diseases [ 41 ]. Exposure to PM in particular has been associated with hyperhomocysteinemia [ 42 , 43 ], which is related to decreased availability of methyl donors; this, in turn, signifies potential decreased global DNA methylation content [ 44 ]. PM is also known to increase the production of reactive oxygen species [ 45 ], which may damage DNA and thus interfere with methylation [ 46 ]. The result may be the hypomethylation of cytosine residues at CpG sites and modification of the expression of genes regulated by DNA methylation [ 47 ], ultimately disrupting transcriptional balance.

In a study of blood leukocyte DNA methylation among elderly male individuals in the Boston area, Baccarelli *et al* . [ 48 ] found that LINE-1 methylation decreased after exposure to PM _2.5_ and black carbon, a tracer for traffic pollution. However, Alu methylation did not appear to be significantly altered. Tarantini *et al* . [ 41 ] also reported a decrease in LINE-1 methylation among male workers in a steel production plant in Brescia, Italy, upon exposure to black carbon. Additionally, LINE-1 and Alu methylation decreased following exposure to PM _10_ . In a crossover trial using concentrated ambient particles (CAPs) to simulate air pollution fluctuations in PM levels, Bellavia *et al* . [ 49 ] found that, although LINE-1 methylation did not significantly change, Alu methylation was decreased following exposure to fine CAPs (<2.5 µm in aerodynamic diameter).

While earlier research has targeted repetitive elements such as LINE-1 and Alu, more recent work has been focused on specific genes, such as the inducible nitric oxide synthase ( *iNOS* ) gene. Increased *iNOS* expression is associated with cardiovascular disease and lung cancer, both conditions also associated with PM exposure [ 50 , 51 ]. In addition to studying LINE-1 and Alu methylation, Tarantini *et al* . [ 41 ] examined the effects of PM _10_ on *iNOS* promotor methylation. While *iNOS* promotor methylation was significantly decreased after 3 days of work, there was no significant association with PM _10_ exposure. Bellavia *et al* . [ 49 ] found no significant effect of CAPs on *iNOS* methylation but reported an association between decreased Alu methylation and increased diastolic blood pressure and significant effects of fine and coarse (2.5–10 µm in aerodynamic diameter) CAPs on increased systolic blood pressure. The authors posited that the lack of observable effect of CAPs on *iNOS* methylation may be due to the smaller number of CpG sites measured in the *iNOS* gene compared to that in Alu rather than a real lack of effect. Although further research is needed to clarify this association, the observed effects suggest that the methylation status of individual genes may change upon exposure to air pollution and subsequently affect the development of disease processes.

Gene-specific alterations to DNA methylation are especially plausible given that smoking appeared to be associated with DNA methylation of the aryl hydrocarbon receptor repressor in several different cohorts. Specifically, one CpG site (cg05575921) has been found to be less methylated among adult smokers [ 52 ] and offspring of women who smoked in pregnancy [ 53 ]. As the concentrations of chemicals are lower in air pollution exposure compared to those in smoking exposure, large sample sizes and/or highly expressed populations will likely be required to identify gene-specific alterations that result from air pollution.

#### Histone Modifications

Histone modifications, along with noncoding RNA expression, have been studied to a lesser extent in the examination of the link between air pollution and epigenetics. Histone modifications occur through processes including acetylation, methylation, phosphorylation, sumoylation, glycosylation, and ADP-ribosylation [ 54 ], with the first two mechanisms being the most common [ 55 ]. In bronchial epithelial cells exposed to diesel exhaust particles, histone deacetylase 1 exhibited degradation while histone acetyltransferase p300 showed increased activation [ 56 ]. Combined with the increased acetylation of histone H4 in the promotor site of cyclooxygenase-2, a gene involved in inflammatory processes, these histone modifications appear to have led to an inflammatory response in the bronchial epithelial cells upon exposure to PM [ 57 ].

#### Noncoding RNA Expression

Among several classes of noncoding RNAs—RNAs that are not translated into proteins but are involved in regulating gene expression both pre- and post-transcriptionally, miRNAs, approximately 22 nucleotides in length [ 58 ], are most widely studied. By base pairing with the 3’-untranslated region of mRNA, miRNA controls the expression of protein-coding genes; this generally results in the inhibition of protein synthesis through either deadenylation of the mRNA transcript or translational repression [ 59 ]. Changes in miRNA expression may occur after both long-term and short-term exposure to air pollution. In a study of rats exposed to ETS for 28 days, exposed rats displayed changes in the expression of miRNA in their lungs, including downregulation of miRNA that had acted as tumor suppressors [ 60 ]. Chemopreventive agents partially mitigated these changes in miRNA expression but did not lead to normal miRNA expression [ 61 ], suggesting the direct involvement of changed miRNA expression in cancerous processes. However, 1 week following cessation of exposure to ETS, some miRNAs, initially downregulated upon ETS exposure, increased in expression, indicating that the effects were reversible to some degree [ 62 ]. This provides evidence for the active role of ETS in triggering changes in miRNA expression.

Additionally, epigenetic changes may occur fairly rapidly instead of requiring longer-term exposure. In a study on the effects of diesel exhaust particles on the expression of miRNAs in differentiated human bronchial epithelial cells cultured at air–liquid interface, researchers observed changes in miRNA expression, both upregulation and downregulation, at as early as the 24-h time point. Bioinformatics analysis also showed that most of these changes appeared to involve the regulation of inflammatory processes [ 63 ]. Jiang *et al* . [ 64 ], in a double-blind crossover study of 16 nonsmoking asthmatic individuals, found in circulating mononuclear cells decreased methylation of genes related to oxidative stress and inflammation, including *GSTP1* , as well as significant changes in the methylation of LINE-1, Alu, and certain miRNAs, at 6 and 30 h following exposure to diesel exhaust. As asthma stems from chronic inflammation, these findings point toward a link between epigenetic changes and inflammation-associated diseases.

Interestingly, viral miRNA may play a role in inducing inflammation along with human miRNA. Viral miRNA in human cells, including Epstein-Barr virus miRNA, Kaposi’s sarcoma-associated herpes virus miRNA, and human immunodeficiency virus-like miRNA [ 65 ], may originate from active infections, or, more commonly, from retrovirus sequences inserted into the host DNA [ 66 ]. In a study of truck drivers and office workers in Beijing, China, Hou *et al* . [ 65 ] found that exposure to traffic elemental carbon for 1 to 2 weeks had significant effects on both human and viral miRNA expression. Expression of viral miRNA appears to affect the expression of other human miRNAs that mediate processes including B cell proliferation, inflammation, and leukocyte recruitment, further exacerbating inflammatory processes. Taken together, these findings provide evidence for both the chronic and acute effects of air pollution in inducing epigenetic changes involved in inflammatory disease processes.

### Epigenetics and Preterm Birth

Building upon the findings on (i) air pollution and preterm birth and (ii) air pollution and epigenetics, investigators have more recently begun to explore the connection between epigenetics and preterm birth. Researchers have focused on DNA methylation and, to a lesser extent, miRNA expression as potential factors contributing to preterm birth. Currently, there are no studies on histone modifications and preterm birth. The relatively small number of studies and their limitations pose challenges to drawing conclusive interpretations; however, existing data are encouraging and warrant further research.

#### DNA Methylation

Researchers have used a variety of media to examine the potential association between epigenetics and preterm birth, the most common of which is blood [ 67 ]. Using umbilical cord blood, Liu *et al* . [ 68 ] examined the association between DNA methylation of imprinted genes and preterm birth. They also studied the effects of DNA methylation on infection status (chorioamnionitis or funisitis), an inflammatory process and risk factor for preterm birth. There was no significant association between DNA methylation and preterm birth; however, increased DNA methylation was associated with infection, suggesting the indirect effect of DNA methylation on preterm birth through infection and related inflammation.

In contrast, other investigators found a signification association between DNA methylation and preterm birth. Again using umbilical cord blood, both Cruickshank *et al* . [ 69 ] and Parets *et al* . [ 70 ] identified CpG sites across the genome associated with preterm birth. Burris *et al* . [ 71 ] analyzed LINE-1 methylation using maternal blood and umbilical cord blood. In the first trimester, lower LINE-1 methylation in maternal blood was associated with preterm birth; however, the opposite was true for cord blood, where higher LINE-1 methylation was found among preterm infants compared to term infants. Additionally, Burris *et al* . [ 72 ], using a gene-specific study design, showed that DNA methylation of a specific aryl hydrocarbon receptor repressor region (different from the region examined in the smoking studies previously mentioned) was more highly methylated among preterm versus term infants.

Investigators have used other tissues as well to study the association between epigenetics and preterm birth. Kim *et al* . [ 73 ] and Maccani *et al* . [ 74 ] studied DNA methylation of CpG sites in the placenta, while Mitsuya *et al* . [ 75 ] utilized myometrial tissue. All three teams observed differential methylation of genes among women who had preterm birth versus those who had term birth. Burris *et al* . [ 27 ], using cervical samples collected between 16 and 19 weeks of gestation, found that increased methylation of LINE-1 was associated with preterm birth. In contrast, the methylation of prostaglandin E receptor 2 ( *PTGER2* ) was inversely associated with preterm birth. Methylation of both LINE-1 and *PTGER2* was associated with evidence of inflammation on Papanicolaou smear, lending further support to the idea of epigenetic involvement in preterm birth via inflammatory processes.

#### MiRNA Expression

MiRNA expression is another epigenetic mechanism that may affect preterm birth. Within a subset of the same cohort of women studied by Burris *et al* . [ 27 ], Sanders *et al* . [ 76 ] analyzed cervical samples gathered between 16 and 19 weeks of gestation. They found associations between the expression of six miRNAs and gestational age at the time of delivery—specifically, increased expression of miRNA was associated with shorter gestation. One of the key targets of these miRNAs is tumor necrosis factor (TNF), which is associated with inflammation. The link between TNF and inflammation is supported by the elevated levels of TNF found in amniotic fluid from preterm labor [ 77 ]. As such, increased miRNA expression may lead to TNF-induced inflammation and thus increase the risk of preterm birth [ 77 , 78 ].

Elovitz *et al* . [ 58 ] analyzed cervical tissue as well and similarly found differential miRNA expression among women who had preterm birth versus those who had term birth. The researchers proposed the following pathway: inflammatory triggers cause miRNA expression to increase, which, in turn, downregulates gene expression. Affected genes may include those coding for proteins involved in cervical remodeling. Thus, changes in miRNA expression may decrease the integrity of the epithelial barrier of the cervix and allow water, inflammatory factors, and other disruptors into the cervix. Interference with cervical remodeling may increase the risk of preterm birth, as short cervical length is a major risk factor for preterm birth.

#### Limitations of Epigenetic Studies of Preterm Birth

Although current findings appear to support an association between epigenetics and preterm birth, several issues complicate the interpretation of study results. First, there are relatively few studies on this topic; the limited body of evidence is not sufficient to conclusively establish an association. Study populations are usually small, and studies may thus lack precision and power to detect an association. Additionally, investigators typically analyze certain genes; however, epigenetic changes may occur genome wide. Furthermore, like studies on air pollution and preterm birth, studies on epigenetics and preterm birth may be vulnerable to misclassification: studies may not differentiate between different types of preterm birth, which may weaken, or even miss, the associations.

The timing of the collection of samples may also affect the findings. Most studies collect samples at the time of delivery. The cross-sectional nature of these samples precludes observation of epigenetic changes throughout the duration of the gestation period [ 67 ]. The importance of time windows may be seen in the study conducted by Burris *et al* . [ 71 ]: LINE-1 methylation during the first trimester, but not the second trimester, was associated with preterm birth. The low correlation between the LINE-1 methylation of the two trimesters suggests that different processes occurred during these intervals; epigenetic processes appear to vary in their effects over the course of gestation. Thus, cross-sectional collection of samples may cause investigators to miss epigenetic effects during specific windows of gestation and prevent the differentiation of causal epigenetic mechanisms from markers of reverse causation.

In addition, the type of sample examined differs between studies. Current studies have utilized blood and placental, myometrial, and cervical tissue samples. Unlike epigenetic studies of air pollution, where interrogating the epigenetics of blood DNA appears to be the logical choice due to the direct alveolar-vascular interface and resultant systemic inflammation from pollution, epigenetic studies of preterm birth do not point to an optimal type of sample [ 67 ]. For example, although Burris *et al* . [ 71 ] found an association between decreased LINE-1 methylation and increased risk of preterm birth using maternal blood obtained during the first trimester, they observed the opposite effect when using cervical tissue [ 27 ]. This discrepancy may be due to the tissue specificity of DNA methylation. As such, comparison across studies that utilize different types of samples may be difficult, as the association may differ depending on the type of sample.

Furthermore, most of these studies did not include cell sorting. Thus, different epigenetic marks may reflect different cell populations, including those from leukocyte invasion, among preterm and term deliveries. Indeed, epigenetic marks may simply reflect differences in local or systemic immune response. Future studies will need to address this ongoing inherent limitation of epigenetic investigations.

However, even in the setting of such limitations, human epigenetic studies are critical. Animal models remain inadequate for the study of preterm birth, as spontaneous preterm birth is rare in most species; additionally, both placentation and labor in humans differ from those in rodents and non-human primates [ 79 ]. As such, human epigenetic studies are integral to furthering understanding of the association between epigenetics and preterm birth.

## Future Directions

Future studies on epigenetics and preterm birth may take various measures to address these limitations and expand current efforts. First, large study cohorts, though more costly and labor-intensive to assemble and follow up on, may increase precision and power to detect an association. Second, expansion of epigenetic mechanisms and types of tissues studied may help deepen understanding and aid in identification of epigenetic markers. Possible endeavors include study of DNA methylation across the genome, examination of noncoding RNAs beyond miRNAs, further work on histone modifications, and analysis of correlations between epigenetic changes and gene expression. Additionally, as with air pollution studies, advanced statistical methods such as distributed lag models may assist with the identification of windows of susceptibility throughout gestation.

Collaboration with other research teams may increase effectiveness. Meta-analyses provide not only opportunities for comparison of results across studies but also chances for identification of patterns in findings and previously undetected associations [ 80 ]. However, meta-analyses are not without their limitations—in particular, variations in study design and laboratory processing of samples may introduce heterogeneity and batch effects and may ultimately dilute effect estimates. Thus, collaboration between epidemiologists and laboratory-based teams in examining the associations between air pollution and cellular changes *in vitro* and in animal models is critical in establishing causal links between air pollution and disease.

## Conclusion

Preterm birth, a key contributor to infant mortality and morbidity and a potential cause of adult chronic disease, remains a major health concern. Air pollution has been implicated as a risk factor for preterm birth, but the literature continues to be inconclusive due to issues such as misclassification from errors in exposure and outcome assessment and heterogeneity between studies. However, biological plausibility lends support to the association between air pollution and preterm birth, and the relatively new field of epigenetics has demonstrated potential pathways through which air pollution may exert its effects. Inflammation appears to play an important role in epigenetic mechanisms across many pathophysiologic states including preterm birth. Whether epigenetic changes lead to persistent inflammatory states—thus increasing the risk for preterm birth—or whether epigenetic mechanisms are triggered by inflammation themselves and go on to affect cellular functions associated with preterm birth is yet to be determined. Although the relatively limited body of literature on epigenetics and preterm birth precludes the establishment of a conclusive association, the existing data are encouraging. Further exploration of this field may enable identification of epigenetic markers, allowing for earlier detection of women at risk for delivering preterm and development of novel therapeutics for the prevention of preterm birth.
